# MM-WAE: Multimodal Wasserstein Autoencoders for Semi-Supervised Wafer Map Defect Recognition

**DOI:** 10.3390/mi17030367

**Published:** 2026-03-18

**Authors:** Yifeng Zhang, Qingqing Sun, Ziyu Liu, David Wei Zhang

**Affiliations:** 1 School of Microelectronics, Fudan University, Shanghai 200433, China; yeefeng@leadyo.com (Y.Z.);; 2 Guangdong Leadyo IC Testing Co., Ltd., Dongguan 523000, China

**Keywords:** wafer map defect recognition, multimodal Wasserstein autoencoder, semi-supervised learning, class imbalance

## Abstract

Wafer map defect pattern recognition is a key task for ensuring yield in integrated circuit manufacturing. However, in real production lines it commonly suffers from scarce labeled data, long-tailed class distributions, and limited feature representations, which cause existing deep learning models to degrade in performance, particularly for minority defect classes and complex defect morphologies. To address these challenges, we propose a semi-supervised classification method for wafer maps based on a multimodal Wasserstein autoencoder (MM-WAE). The framework constructs three parallel feature branches in the spatial, frequency, and texture domains, using a multi-head attention mechanism and gating mechanism for adaptive multimodal fusion. This allows defect patterns to be comprehensively characterized by macroscopic geometric distributions, spectral periodic structures, and microscopic texture details. The Wasserstein autoencoder is introduced, with the latent space distribution regularized by a maximum mean discrepancy (MMD) loss using an inverse multiquadratic kernel. Additionally, an inverse class-frequency weighted cross-entropy loss and a modality consistency loss between the encoder and classifier jointly optimize the reconstruction and classification paths while leveraging large amounts of unlabeled wafer maps for semi-supervised learning. Experimental results show that MM-WAE mitigates performance limitations caused by insufficient labels and class imbalance, significantly improving the accuracy and robustness of wafer defect classification, with promising potential for industrial application and further development.

## 1. Introduction

As semiconductor technology nodes scale down to 5 nm and beyond, wafer fabrication processes are growing increasingly complex, with even minor defects potentially leading to the scrapping of an entire wafer. Wafer map defect pattern recognition (WMDPR) analyzes spatial distribution patterns in wafer bin maps (WBMs), providing essential insights for process diagnosis and yield optimization [1]. Therefore, the development of highly accurate and robust automated defect recognition algorithms is crucial for advancing intelligent wafer fabrication facilities [2,3].

**Figure 1:**
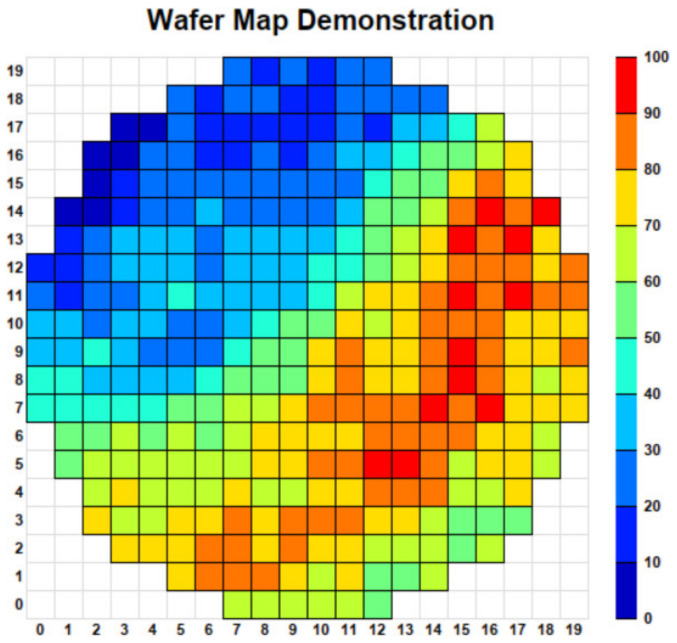
Illustration of a wafer map representation. Each grid cell corresponds to a die location or sampling point on the wafer surface, and the color bar indicates the magnitude of the measured metric.

A wafer map is a two-dimensional representation of the spatial distribution of measurement values or defect indicators across a semiconductor wafer. Each grid cell corresponds to an individual die location or sampling position on the wafer surface, and the color value represents the magnitude of a specific metric, such as yield, defect density, or electrical performance. Figure 1 illustrates a typical wafer map representation, where spatial variations in the metric across the wafer can be visually observed. In practical manufacturing environments, such spatial distributions often reveal process-related defect patterns, including localized clusters, ring-shaped structures, or gradient variations across the wafer surface. These structural characteristics make automated wafer defect pattern recognition a challenging task.

Traditional approaches rely on engineers to visually inspect wafer maps, a time-consuming and subjective process ill-suited to the large data volumes and real-time diagnosis requirements of modern wafer fabs. With the rise of deep learning, many studies have applied convolutional neural networks (CNNs), attention mechanisms, and generative models to automate wafer map classification, achieving high accuracy on benchmark datasets [4,5]. However, deploying these methods in industrial settings presents three main challenges: (1) Labeled data are scarce due to the proprietary nature of wafer maps, limiting the availability of labeled samples. (2) Class imbalance is significant, with normal wafers (class None) vastly outnumbering defective ones, while minority classes like Near-full and Scratch contribute minimally to the dataset. Without proper handling, deep models tend to favor majority classes, undermining detection of critical minority defects. (3) Existing methods often rely on single-channel 2D images for feature extraction in the spatial domain, which fails to capture periodic responses in the frequency domain or subtle texture variations, making spatial features insufficient for robust defect discrimination under noise and process variation [6].

Existing studies have mainly focused on multi-scale and multi-feature fusion within a single wafer image modality. Some methods combine deep features with hand-crafted descriptors (e.g., Radon transform, image moments, statistical features) or jointly train on real wafer maps and samples generated by generative models. These approaches typically perform shallow fusion at the modality level, using feature concatenation or channel-wise weighting, with limited explicit modeling of cross-modal interactions (e.g., between spatial and frequency domains, or between image data and process parameters). Although generative latent-space models, such as autoencoders and variational autoencoders (VAEs), have been explored to address these challenges, they struggle with complex defect patterns and class imbalance. While VAEs reconstruct wafer maps unsupervised and capture global structural information, they fail to model intricate defect patterns fully. Specifically, class imbalance in wafer maps results in poor decision boundary alignment, hindering minority defect detection and affecting model stability.

We propose a WAE-based multimodal framework for wafer map defect pattern recognition. Unlike VAEs, which use Kullback–Leibler (KL) divergence for regularization, WAEs minimize the Wasserstein distance between the aggregated posterior of the latent variables and the prior distribution, resulting in smoother latent geometry and clearer class boundaries. This regularization improves training stability, making WAEs particularly well-suited for handling complex defect patterns and multi-source information fusion tasks. The multimodal Wasserstein autoencoder (MM-WAE) integrates multimodal features and leverages semi-supervised learning to improve defect classification accuracy and robustness, offering promising potential for industrial applications. Experimental results on the WM811K dataset show that MM-WAE significantly outperforms baselines, achieving substantial improvements in macro-averaged precision, recall, and *F*1-score.

The main contributions of this paper are summarized as follows:

(1) We propose a multimodal feature extraction and fusion framework for wafer maps, incorporating three parallel branches in the spatial, frequency, and texture domains to capture geometric shapes, spectral patterns, and surface texture, respectively. A multi-head attention mechanism with gated weighting enables adaptive cross-modal fusion.
(2) We design a WAE-based semi-supervised architecture for latent-space modeling and joint classification. The fused multimodal features are regularized using a Wasserstein autoencoder and a maximum mean discrepancy (MMD) penalty with an inverse multiquadratic kernel to align the latent distribution with a Gaussian prior. A multi-branch classifier in the latent space jointly optimizes reconstruction quality and discriminative performance.
(3) To address wafer map characteristics, we propose a multi-objective training strategy that integrates reconstruction loss, distribution-matching loss, class-frequency weighted cross-entropy loss, and modality-consistency loss, optimizing reconstruction, distribution alignment, and imbalance handling in a unified framework.
(4) We empirically validate the model on the WM811K dataset, showing that MM-WAE significantly outperforms baselines using single-modal features or standard autoencoders, with substantial improvements in macro-averaged precision, recall, and *F*1-score.

The remainder of this paper is organized as follows. Section 2 reviews related work on wafer map defect recognition, semi-supervised learning, and multimodal feature fusion. Section 3 presents the proposed Multimodal Wasserstein Autoencoder (MM-WAE) framework, including multimodal feature extraction, latent-space modeling, and the multi-objective training strategy. Section 4 describes the experimental setup and reports the evaluation results on the WM811K dataset, including comparisons with baseline methods and ablation studies. Finally, Section 5 concludes the paper and discusses the limitations of the present study as well as potential directions for future research.

## 2. Related Work

### 2.1. Wafer Map Defect Recognition

Early methods for wafer map defect pattern recognition relied on handcrafted features, such as Radon transform, geometric shape descriptors, or image moments. These methods require domain expertise and complex feature engineering for satisfactory performance. However, their generalization and robustness are limited when defect morphologies exhibit complex characteristics like multi-scale structures, non-rigid deformations, or composite patterns. With deep learning, convolutional neural networks (CNNs) have become the mainstream for wafer defect recognition. On public datasets like WM811K, researchers have used multi-layer CNNs, ResNets, DenseNets, and related architectures for end-to-end classification of typical defect patterns, achieving higher accuracy than traditional methods [7].

### 2.2. Semi-Supervised Learning for Wafer Defect Recognition

Due to the high cost of annotation and severe class imbalance, semi-supervised learning has gained attention in this domain. Li et al. proposed a ‘manual annotation + semi-supervised iterative relabeling’ framework on the WM811K dataset, improving label quality and recognition performance without large annotation costs, demonstrating the effectiveness of semi-supervised frameworks in imbalanced industrial settings [8]. To address insufficient annotations and skewed data distributions, autoencoders and their variants are widely used in defect detection and classification. Yu et al. proposed SCSDAE, a stacked convolutional sparse denoising autoencoder that learns robust latent representations through unsupervised reconstruction loss, followed by a discriminative head for supervised fine-tuning, improving performance under noise [9]. Wang et al. proposed VAEDLM, an encoder–decoder architecture based on variational autoencoders (VAEs) for generative modeling of defect patterns. Their model outperforms conventional CNNs and GAN-based data augmentation approaches in accuracy, recall, precision, and *F*1-score [10]. Lee and Kim proposed a semi-supervised multi-label learning framework that combines VAEs with CNNs to model labeled and unlabeled wafer maps under mixed-defect scenarios [11].

### 2.3. Multimodal and Multi-Scale Feature Fusion

To improve robustness under noisy conditions, subtle inter-class differences, and severe class imbalance, recent work has begun to explicitly focus on multi-scale and multi-feature fusion. Represented by MFFP-Net, Chen et al. proposed a multi-feature fusion perception network that introduces specially designed fusion modules, enabling downstream layers to extract discriminative features at different scales within a unified high-dimensional representation [12]. This model achieves high classification accuracy and good generalization capability on the real WM-811K dataset. Furthermore, Biswas et al. incorporated global average pooling, parameter reduction, and combinations of convolutional kernels with multiple receptive fields into the network architecture, thereby fusing spatial information at different scales at the feature level [13,14]. Such designs substantiate, from an architectural perspective, the crucial role of multi-scale feature aggregation in enhancing the representational capacity and robustness of wafer defect recognition models.

Despite recent progress, existing methods in wafer map defect recognition face three main challenges: (1) reliance on single-modality spatial features, limiting the capture of complex spatial–frequency characteristics [15]; (2) simplistic multimodal fusion (e.g., concatenation or weighting) with poor modeling of inter-modal correlations; (3) underutilization of unlabeled wafer maps in semi-supervised learning, resulting in limited generalization to rare defects. To address these, we propose the Multimodal Wasserstein Autoencoder (MM-WAE), which integrates spatial, frequency, and texture features through multimodal fusion and latent-space modeling, using reconstruction loss and MMD-based distribution matching to improve classification accuracy and rare defect recognition under label-scarce conditions [16,17].

## 3. Methodology

**Figure 2:**
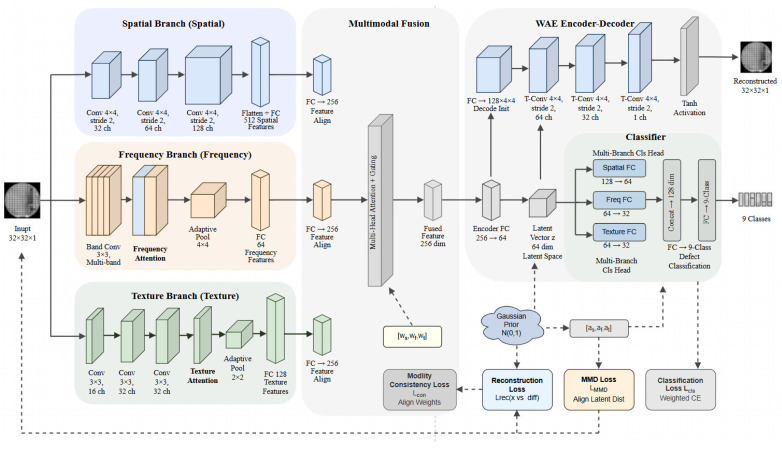
Framework of the Multimodal Wasserstein Autoencoder.

This section introduces the proposed Multimodal Wasserstein Autoencoder (MM-WAE) framework for wafer map defect recognition, as shown in Figure 2. We first define the problem and input representation, followed by the architecture of the model, which integrates multimodal feature extraction and fusion. The core components, including the semi-supervised learning strategy and multi-objective loss function, are also presented.

### 3.1. Problem Definition and Input Representation

Wafer map defect classification aims to automatically identify defect patterns in wafer maps with complex backgrounds and diverse morphologies. A raw wafer map is represented as a single-channel real-valued matrix *x* ∈ ℝ^*H*×*W*^, where *H* and *W* denote the height and width of the wafer map. All wafer maps are resized to 32 × 32 pixels, with pixel intensities normalized to the interval [−1, 1].

The labeled dataset is denoted by $D_{l}=\left\{\left(x_{i},y_{i}\right)\right\},\;i\in \left\{1,\ldots N_{l}\right\}$, where *x_i_* is the *i*-th wafer map sample and $y_{i}\in \left\{1,\ldots ,C\right\}$ represents its corresponding defect class. The unlabeled dataset, containing only wafer images, is denoted by $D_{u}=\left\{x_{j}\right\},\;j\in \left\{1,\ldots N_{u}\right\}$. In industrial scenarios, $N_{u}\gg N_{l}$ typically holds, indicating that the number of unlabeled wafer maps is much larger than the number of labeled samples.

We formulate wafer map defect recognition as a semi-supervised single-label multi-class classification problem. The objective is to learn a mapping f:x→y, from the image space to class space by jointly exploiting both labeled data $D_{l}$ and unlabeled data $D_{u}$. For a given wafer map sample *x*, the model predicts its defect class y∈{1,…,C}. In this study, the number of defect categories is *C* = 9, corresponding to eight representative wafer defect patterns and one non-defect category.

### 3.2. Multimodal Feature Extraction

Wafer defect patterns often exhibit diverse structural characteristics. Some defects present clear global spatial distributions, such as center clusters or edge rings, while others appear as periodic structures or fine-grained local irregularities. These heterogeneous characteristics make it difficult for a single representation to fully capture the complexity of wafer defect patterns.

To address this issue, the proposed framework extracts complementary feature representations from three perspectives: spatial structure, spectral-aware responses, and texture statistics. These representations describe defect patterns at different structural levels and provide complementary information for defect recognition. The spatial branch focuses on the global geometric layout of defects, the frequency-aware branch emphasizes periodic or structured patterns, and the texture branch captures fine-grained local variations.

#### 3.2.1. Spatial Feature Branch

The spatial branch captures the macroscopic geometric structure and layout distribution of wafer defects. Many defect types in wafer maps exhibit distinctive global spatial patterns, such as central clusters, edge rings, or localized regions. Therefore, modeling the spatial arrangement of defects is crucial for reliable defect recognition.

This branch adopts a three-stage convolutional downsampling architecture with 4 × 4 kernels (stride 2, padding 1), and output channels of 32, 64, and 128. After three convolutional stages, the 32 × 32 input wafer map is mapped to a 4 × 4 × 128 feature map. The feature map is then flattened and passed through a fully connected layer to obtain a 512-dimensional spatial feature vector $f^{\left(s\right)}\in R^{512}$. This lightweight convolutional backbone efficiently extracts global shape and regional distribution features of wafer defects. By explicitly modeling spatial structures, the spatial branch provides robust representations for defect categories characterized by large-scale geometric patterns.

#### 3.2.2. Frequency-Aware Feature Branch

Certain wafer defect patterns exhibit periodic or structured spatial characteristics, such as ring-shaped defects and streak-like patterns. These structures often correspond to distinctive spectral responses in the frequency domain. To enhance the sensitivity of the model to such patterns, a frequency-aware feature branch is introduced. Instead of applying explicit frequency transforms (e.g., FFT or DCT), this branch employs learnable convolutional filters to model localized spectral responses. According to the convolution theorem, spatial convolution corresponds to filtering operations in the frequency domain. Therefore, convolution kernels can implicitly approximate band-pass filtering effects through learned frequency responses.

Specifically, a 3 × 3 convolutional filter bank is applied to generate four feature maps $\left\{F_{k}\right\},\;k=1,2,3,4$. These feature maps capture different spectral responses of the input wafer map. Global average pooling is applied to each feature map to obtain a 4-dimensional vector, which is then passed through a two-layer fully connected network to generate frequency attention weights $\alpha \in R^{4}$, normalized using a Softmax function:

$$\tilde{F_{k}}=\alpha_{k}\cdot F_{k},\;k=1,2,3,4$$

The attention-enhanced feature maps are further processed by 4 × 4 adaptive pooling and flattening to obtain a 64-dimensional feature vector $f^{\left(f\right)}\in R^{64}.$ This frequency-aware mechanism allows the model to emphasize spectral patterns associated with periodic defect structures, improving recognition robustness for defect categories such as Donut and Edge-Ring.

#### 3.2.3. Texture Feature Branch

Unlike traditional handcrafted texture descriptors such as the gray level co-occurrence matrix (GLCM), which rely on manually designed statistical features, the proposed texture branch employs convolutional neural networks to automatically learn hierarchical texture representations from wafer map data. This learning-based approach enables the model to capture complex and irregular texture patterns that are difficult to characterize using handcrafted descriptors.

In wafer maps, certain defect types exhibit subtle local variations, including clustered points, scattered structures, and scratch-like patterns. These fine-grained characteristics are difficult to distinguish using only global spatial features. Therefore, the texture branch is designed to enhance the model’s sensitivity to local structural variations.

Specifically, this branch consists of three 3 × 3 convolutional layers with channel sizes of 16, 32, and 32, respectively, followed by a texture attention module that focuses on texture-salient regions. After attention enhancement, the feature maps are processed by 2 × 2 adaptive pooling and flattened to obtain a 128-dimensional texture feature vector $f^{\left(t\right)}\in R^{128}$. By capturing fine-grained local structures, the texture branch improves the model’s ability to distinguish subtle defect patterns, particularly for categories such as Random, Loc, and Scratch.

#### 3.2.4. Multimodal Feature Fusion

To integrate the complementary information by different feature representations, a multimodal feature fusion module is employed.

First, the three feature vectors are projected into a unified feature space through separate fully connected layers

$${\tilde{f}}^{\left(s\right)}=W_{s}f^{\left(s\right)},\;{\tilde{f}}^{\left(f\right)}=W_{f}f^{\left(f\right)},\;{\tilde{f}}^{\left(t\right)}=W_{t}f^{\left(t\right)},$$

where

$${\tilde{f}}^{\left(m\right)}\in R^{d_{m}},\;d_{m}=256.$$

The projected features are stacked to form a sequence:

$$F={\left[{\tilde{f}}^{\left(s\right)},{\tilde{f}}^{\left(f\right)},{\tilde{f}}^{\left(t\right)}\right]}^{T}\in R^{3\times d_{m}}.$$

A multi-head self-attention (MHA) module is applied to model interactions among different feature representations:

$$F^{\prime }=MHA\left(F\right)+F.$$

The residual connection preserves the original representation while enabling cross-domain interactions. After obtaining the interaction-enhanced features, a gated fusion network learns the importance weights of each feature representation:

$$w=Softmax\left(W_{g}\left[{\tilde{f}}^{\left(s\right)};\;{\tilde{f}}^{\left(f\right)};\;{\tilde{f}}^{\left(t\right)}\right]\right).$$

The final fused feature vector is computed as:

$$f=\sum_{m\in \left\{s,f,t\right\}}w_{m}{\tilde{f}}^{\left(m\right)}\in R^{d_{m}}$$

**Figure 3:**
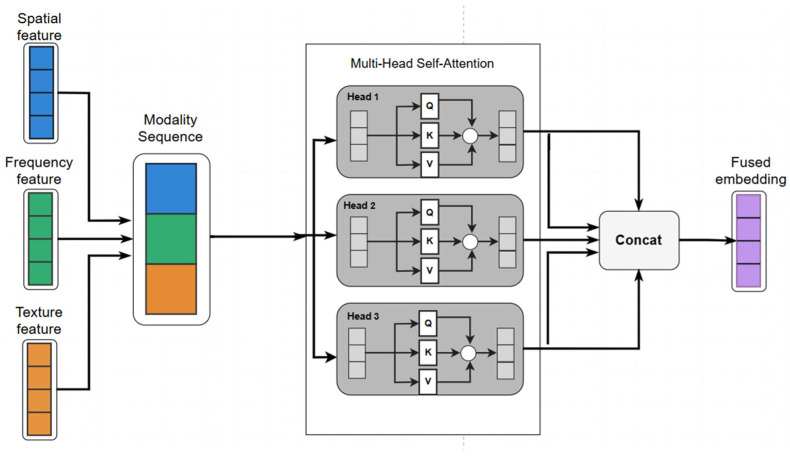
Schematic of the Multi-head Attention Mechanism.

This adaptive fusion mechanism enables the model to dynamically balance spatial, spectral-aware, and texture information according to the characteristics of the input wafer map, thereby improving robustness in recognizing diverse defect morphologies, as shown in Figure 3.

### 3.3. Multimodal Wasserstein Autoencoder Architecture

To further improve representation quality, the fused multimodal feature vector is modeled in a structured latent space using a Wasserstein autoencoder (WAE). The WAE framework maps the fused feature representation into a low-dimensional latent space, reconstructs the input features, and simultaneously learns discriminative latent representations for classification.

Compared with conventional variational autoencoders (VAEs), which rely on KL-divergence to regularize the latent distribution, WAEs align the aggregated posterior distribution with a predefined prior using distribution-matching techniques such as Maximum Mean Discrepancy (MMD). This formulation allows more flexible control of the latent distribution and often leads to smoother and more stable latent representations.

#### 3.3.1. Latent Space Mapping

The fused feature vector $f\in R^{256}$ is mapped to a 64-dimensional latent space through a linear transformation:

$$z=W_{z}f+b_{z},\;z\in R^{64}.$$

The latent vector *z* captures compact structural representations of wafer defect patterns and serves as the shared representation for both reconstruction and classification tasks.

#### 3.3.2. Reconstruction Decoder

The decoder reconstructs the wafer map representation from the latent vector. First, the latent vector *z* is projected into a high-dimensional feature map:

$$h_{0}=\Phi (W_{d}z+b_{d}),\;h_{0}\in R^{128\times 4\times 4},$$

where Φ(·) denotes the ReLU nonlinear activation function. Three transposed convolution layers are then applied for upsampling, using 4 × 4 kernels (stride 2, padding 1) with output channels of 64, 32, and 1, respectively. Finally, a Tanh activation function constrains the reconstructed output within the range [−1, 1], yielding the reconstructed wafer map.

This decoder design balances reconstruction accuracy and parameter efficiency, which is particularly suitable for semi-supervised learning settings where the encoder features are shared with a classification network.

#### 3.3.3. Multimodal Classifier

The classification module utilizes the latent representation while preserving modality-specific information through a multi-branch classifier. Each branch receives the latent vector *z* and applies a two-layer fully connected network to produce modality-specific representations: $g^{\left(s\right)}(z),g^{\left(f\right)}(z),g^{\left(t\right)}(z)$.

A learnable branch-weight vector $a=\left[a_{s},a_{f},a_{t}\right]$, is normalized by a Softmax function to compute the importance of each representation branch. The weighted representations are concatenated to form a 128-dimensional feature vector $h^{\left(c\right)}$. The final class score vector is computed as:

$$s=W_{c}h^{\left(c\right)}+b_{c},\;s\in R^{9}$$

The predicted class probability is obtained using:

$$p=Softmax(s),\;p\in R^{9}.$$

In addition, a consistency constraint is applied between the modality weights learned in the encoder-side fusion module and the classifier-side branch weighting mechanism. This regularization encourages coherent multimodal representation learning and improves classification stability.

### 3.4. Multi-Objective Loss Function

**Figure 4:**
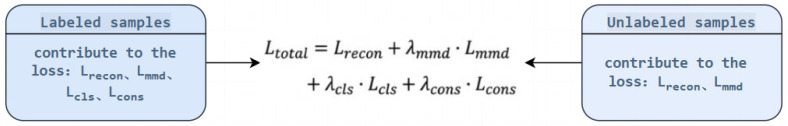
Loss Function.

This work designs a multi-objective loss function to optimize reconstruction quality, latent distribution alignment, classification performance, and modality weight consistency. The loss formulation is shown in Figure 4.

#### 3.4.1. Reconstruction Loss

The reconstruction loss measures the discrepancy between the input wafer map and its reconstructed output. Given a mini-batch of size *N*, the reconstruction loss is defined as

$$L_{recon}=\frac{1}{N}\sum_{i=1}^{N}{\left\|x_{i}-{\hat{x}}_{i}\right\|}_{1}$$

where $x_{i}$ denotes the input wafer map and ${\hat{x}}_{i}$ represents the reconstructed output generated by the decoder.

Compared with the *L*_2_ loss, the *L*_1_ loss is less sensitive to outliers and better preserves structural details in reconstructed images, which helps produce more stable reconstructions under noisy conditions.

#### 3.4.2. Distribution Matching Loss (MMD)

To align the latent vector distribution with the standard normal prior p(z)=N(0,I), we use maximum mean discrepancy (MMD) with an inverse multiquadratic kernel. Let ${\left\{z_{i}\right\}}_{\;i=1}^{\;\;N}$ be the latent vectors from the encoder, and ${\left\{\tilde{z}\right\}}_{\;j=1}^{\;\;M}$ be random vectors sampled from the standard normal prior. The inverse multiquadratic kernel is:

k(u,v)=cc+‖u−v‖22

where *c* > 0 is a constant. The MMD is:

$$L_{mmd}=\frac{1}{N^{2}}\sum_{i=1}^{N}\sum_{j=1}^{N}k(z_{i},z_{j})+\frac{1}{M^{2}}\sum_{i=1}^{M}\sum_{j=1}^{M}k({\tilde{z}}_{i},{\tilde{z}}_{j})-\frac{2}{NM}\sum_{i=1}^{N}\sum_{j=1}^{M}k(z_{i},{\tilde{z}}_{j})$$

Minimizing the MMD aligns the latent-space distribution with the Gaussian prior, improving the continuity and separability of the representations.

#### 3.4.3. Weighted Classification Loss

To address the imbalanced distribution of defect categories, a dynamic class-frequency weighting scheme is added to the classification loss. Let $y_{i,c}$ be the one-hot label for class c, and $p_{i,c}$ the predicted probability. The class weight w_c_ is defined as an inverse function of class frequency π_c_; for example,

$$w_{c}=\frac{1}{\log\left(\alpha +\pi_{c}\right)},$$

where α > 1 as a smoothing constant. The weighted cross-entropy loss is:

$$L_{cls}=-\frac{1}{N}\sum_{i=1}^{N}\sum_{c=1}^{C}w_{c}y_{i,c}\log p_{i,c}.$$

This scheme increases the gradient contribution of minority-class samples, mitigating model bias toward majority classes and improving recognition of rare defects like Scratch and Loc.

#### 3.4.4. Modality Consistency Loss

To ensure consistency between the modality weights www from the encoder and a from the classifier, we introduce a modality consistency loss. Let Δ = w − a be the difference vector between the modality weights. The consistency loss is the sum of the variance and standard deviation of Δ:

$$L_{cons}=Var\left(\Delta \right)+Std(\Delta ).$$

When the encoder and classifier assign similar importance to each modality, fluctuations in Δ decrease, reducing the loss. This encourages consistent cooperation of multimodal information in encoding and decision-making.

#### 3.4.5. Overall Loss

The overall optimization objective is obtained by integrating the four loss terms:

$$L_{total}=L_{recon}+\lambda_{mnd}\times L_{mnd}+\lambda_{cls}\times L_{cls}+\lambda_{cons}\times L_{cons}$$

where $L_{total}$ denotes the overall multi-objective loss, $L_{recon}$ the reconstruction loss, $L_{mmd}$ the distribution-matching loss, $L_{cls}$ the classification loss, and $L_{cons}$ the modality consistency loss. The coefficients $\lambda_{mmd},\;\lambda_{cls},\;\lambda_{cons}$ are preset weighting factors that balance the contributions of the respective objectives.

### 3.5. Semi-Supervised Training Strategy

Since unlabeled samples contain rich structural and distributional information, we adopt a semi-supervised strategy that utilizes both the labeled set $D_{l}$ and the unlabeled set $D_{u}$.

For labeled mini-batches, all four loss terms are computed on samples from $D_{l}$, optimizing both reconstruction and classification. For unlabeled mini-batches, only $L_{mmd}$ and $L_{rec}$ are evaluated on $D_{u}$. This allows the reconstruction and distribution-matching constraints to guide the encoder in learning more discriminative latent representations, without relying on potentially erroneous pseudo-labels. During optimization, we use the Adam optimizer with gradient clipping to prevent gradient explosion.

## 4. Experiments and Results

### 4.1. Dataset and Evaluation Metrics

#### 4.1.1. WM811K Dataset

**Figure 5:**
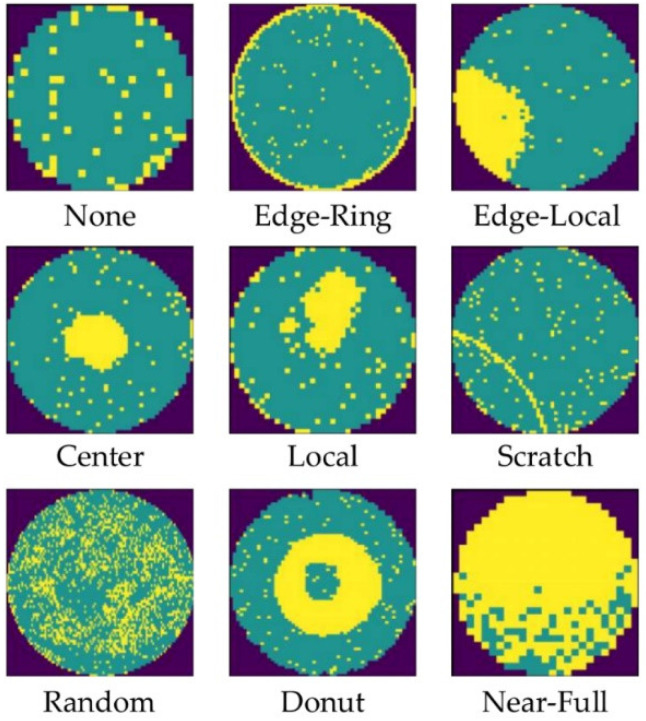
Representative examples of defect samples from the WM811K dataset.

In this study, we evaluate the effectiveness of the proposed method on the publicly available WM811K wafer defect dataset. WM811K, the largest publicly available wafer map dataset, contains 811,457 maps collected from real semiconductor production lines. The dataset includes eight defect patterns (Center, Donut, Edge-Ring, Edge-Loc, Loc, Random, Scratch, Near-Full) and a defect-free category (None). Examples from the dataset are shown in Figure 5.

#### 4.1.2. Evaluation Metrics

To evaluate the performance of the proposed MM-WAE model for wafer defect classification, four widely used evaluation metrics are adopted: precision, recall, *F*1-score, and accuracy.

In the multi-class classification setting, each defect category ccc is treated as a binary classification problem using a one-versus-rest strategy. For each class c, the confusion matrix is constructed, where $TP_{c}$, ${FP}_{c}$, $FN_{c}$, and $TN_{c}$ denote the numbers of true positives, false positives, false negatives, and true negatives, respectively.

***Precision***. Precision measures the proportion of samples predicted as class ccc that truly belong to that class. It reflects the reliability of the model in avoiding false alarms. The precision for class *c* is defined as

$${Precision}_{c}=\frac{TP_{c}}{TP_{c}+FP_{c}}.$$

Higher precision indicates that fewer non-defect samples are incorrectly predicted as defect samples.

***Recall***. Recall measures the proportion of actual class-*c* samples that are correctly identified by the model. This metric reflects the ability of the model to detect defect samples without missing them. The recall is defined as

$${Recall}_{c}=\frac{TP_{c}}{TP_{c}+FN_{c}}.$$

A higher recall indicates fewer missed defect detections.

***F*1-score**. Precision and recall often exhibit a trade-off relationship. The *F*1-score combines them into a single metric using their harmonic mean:

$${F1}_{c}=\frac{2\cdot {Precision}_{c}\cdot {Recall}_{c}}{{Precision}_{c}+{Recall}_{c}}.$$

The *F*1-score provides a balanced evaluation of detection accuracy and completeness, which is particularly useful for datasets with imbalanced defect categories.

***Accuracy***. Accuracy measures the overall proportion of correctly classified samples among all test samples. It is defined as

$$Accuracy=\frac{\sum_{c=1}^{C}{TP}_{c}}{N}.$$

where *N* denotes the total number of samples. Accuracy provides an intuitive indicator of the overall classification performance of the model.

These evaluation metrics are widely adopted in wafer defect recognition studies and provide complementary perspectives for assessing classification reliability, defect detection capability, and overall model performance.

### 4.2. Experimental Setup

#### 4.2.1. Overview of the Experimental Setup

To ensure a rigorous and unbiased evaluation of the semi-supervised MM-WAE framework, we implement a systematic data partitioning and sampling protocol on the WM811K dataset. Starting from a total pool of 172,950 labeled wafers across nine defect categories, the data is initially divided into three disjoint sets: a training set (70%, 121,065 samples), a validation set (10%, 17,295 samples), and a test set (20%, 34,590 samples). To simulate an industrial label-scarce scenario within the training partition, stratified random sampling is applied to select labeled subsets at 5% and 10% ratios, thereby preserving the inherent class imbalance of the original distribution. Table 1 shows the detailed sample distribution across categories and partitions at 5% Labeling Ratio. The remaining training samples, with their labels intentionally discarded, constitute the unlabeled pool. Crucially, to prevent data leakage, we strictly ensure that no samples from the validation or test sets participate in any phase of the training process, including the unsupervised reconstruction and MMD-based latent distribution matching.”

Recognizing that semi-supervised performance—especially at a low 5% labeling rate—can be sensitive to the specific samples chosen for D_l_, we implemented a multi-run validation protocol. All experiments for MM-WAE and the baseline models (Supervised-CNN, Pseudo-Labeling, MixMatch, FixMatch, and MMVAE) were repeated over three independent runs using different random seeds. We report the mean and standard deviation (±σ) for all primary metrics (Accuracy, Macro-Precision, Macro-Recall, and Macro-*F*1). This provides a statistically sound basis to confirm that the performance gains achieved by MM-WAE are robust and not a result of favorable sampling bias.

#### 4.2.2. Comparative Models

To evaluate the semi-supervised performance of MMWAE, we construct five baseline models for comparison:

(1) Supervised-CNN: a purely supervised convolutional baseline. This model uses the same convolutional backbone and classification head as the image branch of MMWAE but removes the encoder–decoder structure and latent distribution constraints. It is trained only on the ρ fraction of labeled samples in $D_{l}$, without using any unlabeled samples from $D_{u}$. Supervised-CNN serves as a reference lower bound for the performance of conventional supervised learning under extremely limited label availability.
(2) Pseudo-Label-CNN: a discriminative pseudo-label semi-supervised baseline, sharing the same architecture as Supervised-CNN. During training, we pretrain an initial classifier on the ρ fraction of $D_{l}$ and use it to predict labels for samples in $D_{u}$. Samples with prediction confidence above a threshold τ are assigned pseudo-labels and added to the labeled training set, with model parameters updated iteratively. Pseudo-Label-CNN is used to evaluate the applicability of classical pseudo-label based semi-supervised strategies to the wafer defect classification task.
(3) MixMatch: An advanced data-driven semi-supervised baseline that utilizes holistic symmetry for label refinement. It employs “label guessing” by averaging predictions from multiple weakly augmented versions of the same unlabeled image, followed by a “sharpening” operation to reduce entropy. Finally, it uses MixUp to interpolate labeled and unlabeled data, creating a continuous decision boundary. MixMatch is used to evaluate the impact of standard data augmentation and interpolation strategies on wafer map defect patterns.
(4) FixMatch: A state-of-the-art consistency-based semi-supervised baseline. It leverages a “weak-to-strong” consistency regularization mechanism: the model generates a pseudo-label from a weakly augmented version of an unlabeled image and uses it to supervise the prediction of a strongly augmented version of the same image, provided the prediction confidence exceeds a high threshold. FixMatch represents the performance ceiling for single-modality spatial feature learning in semi-supervised scenarios.
(5) MMVAE: a multimodal generative semi-supervised baseline, sharing the same convolutional encoder, decoder, and multi-label classification head as MMWAE, and explicitly modeling the joint distribution of spatial, frequency, and texture modalities in the latent space. The main difference from MMWAE is the choice of latent regularization: MMVAE uses the standard VAE Kullback–Leibler (KL) divergence, encouraging the posterior q(z∣x) to contract toward an isotropic Gaussian prior p(z). During training, for labeled samples in $D_{l}$ we optimize the reconstruction loss, KL regularization, and classification loss, while for unlabeled samples in $D_{u}$, we only optimize the reconstruction and KL terms to improve the latent representations.

Aside from structural differences, all four models share identical feature dimensionalities, network depth, number of epochs, optimizer settings, and other hyperparameters, ensuring an objective comparison of latent-space modeling and semi-supervised learning strategies.

#### 4.2.3. Implementation Details

To ensure fairness in the comparative experiments, we use a unified implementation and hyperparameter configuration for training and evaluating the multimodal Wasserstein autoencoder (MMWAE) on the WM811K dataset. All experiments are conducted using the PyTorch (1.13.1) framework on a workstation equipped with dual NVIDIA RTX A5000 graphics processing units (GA102 architecture) sourced from NVIDIA Corporation (Santa Clara, CA, USA), with the server system manufactured by Dell Inc. (Round Rock, TX, USA).

For data preprocessing, we read wafer maps and defect labels from the official WM811K files. Each wafer’s two-dimensional map is retained and resized to 32 × 32 resolution. Pixel values are normalized in two steps: channel-wise min–max normalization to [0, 1], then a linear mapping to [−1, 1]. Each wafer map is represented as a single-channel input tensor.

All experiments use the Adam optimizer with an initial learning rate of 5 × 10^−4^ and weight decay of 1 × 10^−5^. The batch size is 256, and the maximum training epochs are set to 100. An early stopping strategy is used: training stops if validation accuracy does not improve for 30 epochs, and the best model parameters are retained. To improve stability, we apply gradient clipping with a maximum l2-norm of 1.0 during backpropagation.

In the semi-supervised setting, we employ stratified random sampling to select a fraction ρ ∈ {5%, 10%} of the labeled set $D_{l}$ for training, while the remaining labeled samples are treated as unlabeled data in $D_{u}$. Recognizing that the performance of semi-supervised models at such low labeling ratios can be highly sensitive to the initial data selection, we repeat all experiments—including the proposed MM-WAE and all comparative baselines—across three independent runs using different random seeds (42, 123, and 2026). All evaluation metrics on the test set are reported as the Mean ± Standard Deviation (μ ± σ). This multi-seed protocol ensures the statistical reliability of the observed performance gains and mitigates the risk of reporting results based on anomalous samples. For the classification objective, all models utilize standard cross-entropy loss, while generative baselines (MMVAE and MM-WAE) incorporate additional reconstruction and latent regularization terms (KL or MMD). All other structural and training configurations remain identical across models to ensure an objective comparison.

### 4.3. Experimental Results

#### 4.3.1. Results on 5% Labeled Data

Table 2 shows the evaluation metrics of the six models on the WM811K dataset with only 5% of the training samples labeled. To eliminate the bias introduced by random sampling at such a low labeling ratio, all results are reported as the Mean ± Standard Deviation over three independent runs with different random seeds.

With 5% labeled data, Supervised-CNN achieves an accuracy of 82.62 ± 0.74%, with precision and recall at 79.21% and 79.39%, respectively. In this label-scarce setting, its performance is highly limited, especially in generalizing to minority classes and boundary samples. Using a simple pseudo-labeling mechanism and iterative self-training on unlabeled data, Pseudo-Label-CNN improves accuracy to 86.11%. To further evaluate the effectiveness of semi-supervised learning (SSL), we introduced mature SSL baselines. The results show that MixMatch achieves an accuracy of 89.26%, while FixMatch significantly boosts the accuracy to 92.11%. This substantial leap demonstrates that effectively harnessing the 95% unlabeled data natively resolves the label scarcity bottleneck without any data leakage.

In comparison, multimodal generative approaches show even clearer advantages. With 5% labeled data, MMVAE achieves an accuracy of 93.24%, surpassing the state-of-the-art SSL baseline FixMatch by over 1 percentage point. Its *F*1-score reaches 91.32%, indicating that multimodal latent distribution modeling enhances both feature representation and performance on imbalanced classes.

The proposed MM-WAE further pushes the state-of-the-art, achieving an accuracy of 96.11%, with precision and recall at 95.45% and 95.88%, and an *F*1-score of 95.66%. MM-WAE improves accuracy by about 13.5 percentage points over Supervised-CNN, 4 percentage points over FixMatch, and nearly 3 percentage points over MMVAE. Crucially, MM-WAE exhibits the lowest standard deviation (±0.12% to ±0.22% across all metrics), proving its exceptional stability against initial data selection. The consistent trends in precision, recall, and *F*1-score firmly validate that MM-WAE effectively exploits latent structural information through its multimodal branches and Wasserstein regularization.

**Figure 6:**
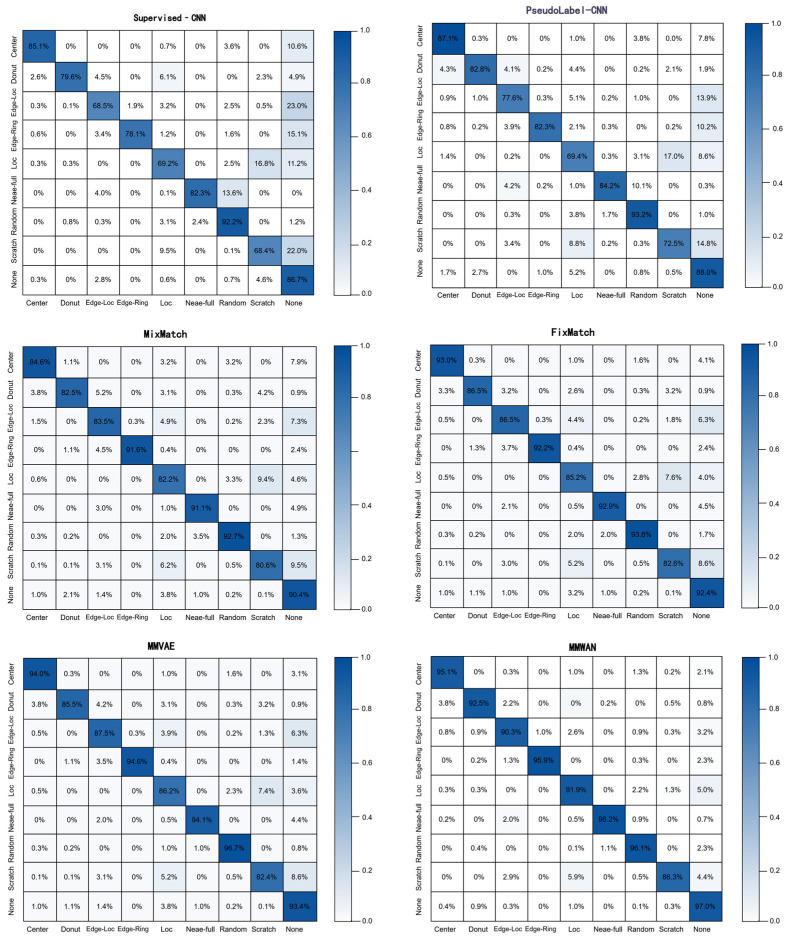
Comparison of confusion matrices for different models under the 5% labeled data setting on the WM811K dataset.

Figure 6 shows the confusion matrices for the evaluated models at the 5% labeling ratio, taking the experimental run with the random seed of 2026 as a representative example. Supervised-CNN and Pseudo-Label-CNN show high diagonal accuracies for majority classes (e.g., Center, Donut) but exhibit noticeable misclassification for defect types with fewer samples and more confusable patterns, like Scratch and Loc. While FixMatch and MMVAE strengthen the diagonal for most classes, MM-WAE shows the strongest diagonal in the confusion matrix, with marked improvements in the classification of minority classes. This suggests that MMD-based Wasserstein autoencoder regularization enables MM-WAE to more effectively capture the latent structure of unlabeled wafer maps, enhancing the discriminability of representations and the robustness of decision boundaries.

#### 4.3.2. Results on 10% Labeled Data

At the 10% labeling ratio (Table 3), the overall performance of all models naturally improves as the number of labeled samples increases. Supervised-CNN’s accuracy rises to 83.12%, with precision and recall at 81.26% and 81.45%. This shows that increasing the labeled data significantly enhances the performance of the supervised model. However, Pseudo-Label-CNN achieves an accuracy of 84.15%, slightly lower than its 5% counterpart, reflecting the sensitivity of standard pseudo-labeling methods to pseudo-label quality: as noise accumulates during iterative training, the benefit does not always increase linearly with the labeling ratio. Conversely, advanced SSL baselines continue to show robust performance, with MixMatch and FixMatch reaching 91.32% and 94.75% accuracy, respectively.

In the 10% labeling scenario, generative multimodal methods maintain their clear supremacy. MMVAE achieves 95.24% accuracy, improving by about 12 percentage points over Supervised-CNN. MM-WAE (Ours) further increases the accuracy to an impressive 97.11%, with precision at 97.11%, recall at 97.23%, and an *F*1-score of 97.17%. Compared to the baselines, MM-WAE gains about 14 percentage points in accuracy over Supervised-CNN, 2.3 percentage points over FixMatch, and nearly 2 percentage points over MMVAE. Moreover, the standard deviation of MM-WAE’s accuracy drops to a mere ±0.12%, highlighting its extreme robustness.

**Figure 7:**
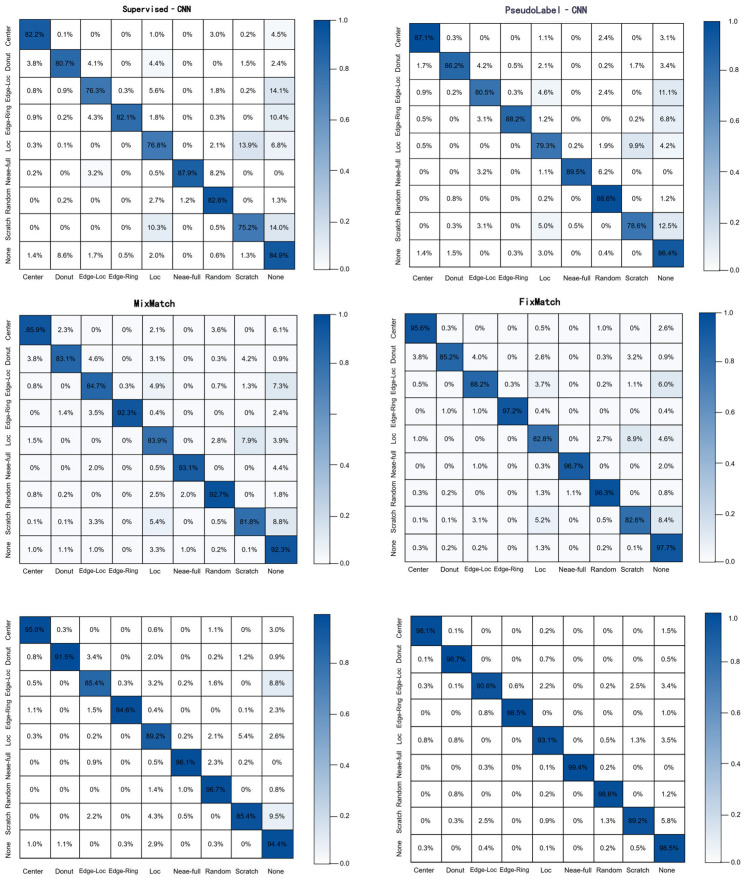
Comparison of the confusion matrices on the WM811K dataset under the 10% labeled data setting.

Figure 7 shows the confusion matrices for the evaluated models at the 10% labeling ratio, taking the experimental run with the random seed of 2026 as a representative example. As the labeling ratio increases from 5% to 10%, MM-WAE improves diagonal accuracy for most defect categories and maintains superior performance for orientation-sensitive, complex, or rare defect patterns.

In summary, MM-WAE consistently outperforms Supervised-CNN, mature SSL baselines (MixMatch, FixMatch), and MMVAE in precision, recall, *F*1-score, and accuracy, especially in the highly label-scarce 5% setting. These results definitively demonstrate that a semi-supervised framework based on multimodal feature fusion and Wasserstein autoencoder modeling can effectively address bottlenecks from limited labeled data and class imbalance, offering significant practical value for automatic defect classification in industrial wafer manufacturing.

#### 4.3.3. Analysis of Computational Efficiency and Model Complexity

The proposed MM-WAE, alongside the MMVAE baseline, involves a total of approximately 1.90 M trainable parameters. While this is an increase compared to the single-modal CNN baselines (approximately 0.56 M), the absolute scale remains significantly smaller than standard deep learning backbones frequently used in computer vision, such as ResNet-18 (~11.18 M) or ResNet-50 (~25.56 M). The moderate increase in parameters is strategically allocated to the parallel frequency and texture branches to capture heterogeneous defect signatures.

A critical advantage of the MM-WAE framework is the asymmetry between the training and inference phases. While the full encoder–decoder architecture (1.90 M parameters) is required during training to regularize the latent space through reconstruction and distribution matching, the Decoder is entirely discarded during deployment. For online inference on the production line, only the multi-modal Encoder and the classification head are utilized. This reduces the effective inference parameter count significantly, enabling low-latency, high-throughput defect diagnosis. Such “slimming” ensures that the system can be deployed on edge computing units with limited resources while maintaining the robust representation capabilities gained from generative modeling.

### 4.4. Ablation Study

To further verify the effectiveness of the proposed multimodal generative modeling design, we conduct a systematic ablation study on the WM811K dataset under the 10% labeled-data setting. We construct several variants of MMWAE with different modality combinations: MMWAE-S uses only the spatial modality; MMWAE-SF adds the frequency-domain modality (Spatial + Frequency); MMWAE-ST adds the texture modality (Spatial + Texture); and the full MMWAE model fuses all three modalities (Spatial + Frequency + Texture). For all four models, the feature dimensionality, network depth, training epochs, and optimizer settings are kept identical to the original MMWAE; only the input modalities and their corresponding encoder/decoder branches vary. These variants enable a quantitative assessment of the contribution of each modality and their combinations to the final performance. The results are summarized in Table 4.

We first use the single-modality MMWAE-S as the baseline and analyze the performance gains from adding a second modality. As shown in Table 4, MMWAE-S achieves a precision of 91.45%, recall of 92.26%, *F*1-score of 91.85%, and accuracy of 93.65%, demonstrating that the generative semi-supervised framework is highly discriminative even in the single-modality setting. Adding the frequency-domain branch to form MMWAE-SF further improves all metrics: precision increases to 93.62%, recall to 93.84%, and *F*1-score and accuracy to 93.73% and 95.63%, respectively, with gains of about 1.88 and 1.98 percentage points over the baseline. This suggests that the frequency-domain modality complements the spatial modality by capturing periodic structures and ring-like patterns, which is particularly beneficial for defect types like Edge-Ring and Donut, and reduces confusion with classes like Center and Random.

In comparison, MMWAE-ST, which adds only the texture modality, achieves a precision of 92.02%, recall of 91.96%, *F*1-score of 91.99%, and accuracy of 94.96%. These values are superior to MMWAE-S, but slightly lower than MMWAE-SF. This indicates that texture features capture fine-grained local patterns, which are useful for representing dot-like and line-like defects like Random and Scratch, but are less effective than frequency-domain features for modeling global structures.

After confirming the effectiveness of dual-modality combinations, we further examine the benefits of tri-modality fusion. The full MMWAE model integrates spatial, frequency, and texture information, achieving the best performance across all four metrics: precision 96.11%, recall 96.23%, *F*1-score 96.17%, and accuracy 97.11%. Compared to the single-modality baseline MMWAE-S, the *F*1-score and accuracy improve significantly by about 4.32 and 3.46 percentage points, respectively; compared to the strong MMWAE-SF, the full model still yields an additional 2.44 and 1.48 percentage point gain in *F*1-score and accuracy. Given the already high performance level, this improvement is substantial.

In summary, the multimodal ablation study shows that the generative semi-supervised framework effectively exploits structural information in unlabeled samples with a single modality, while the frequency and texture modalities provide complementary information on global structure and local details. Their joint modeling with the spatial modality is crucial for long-tail defect classes. The fusion of all three modalities underpins MMWAE’s high performance. The gains stem not from increasing network depth or parameter count, but from incorporating priors on multi-scale, heterogeneous wafer defect patterns and effectively coordinating them at the feature level.

## 5. Conclusions

This work addresses key challenges in wafer defect classification, such as limited labeled data and severe class imbalance, by proposing a semi-supervised multimodal Wasserstein autoencoder (MMWAE). The proposed framework extracts spatial, frequency-aware, and texture features in parallel and fuses them through multi-head attention and gating mechanisms to obtain complementary representations. A Wasserstein autoencoder is adopted to embed the fused features into a structured latent space, and a multi-objective optimization strategy combining reconstruction loss, MMD-based distribution matching, class-weighted cross-entropy, and modality-consistency regularization is used to improve representation learning and classification robustness. Experimental results on the WM811K dataset demonstrate that MMWAE outperforms several baseline methods under low-labeling conditions and effectively exploits unlabeled data for defect recognition. As the labeling ratio increases, the performance of MMWAE approaches that of fully supervised models while maintaining advantages for certain defect categories with complex structures or limited samples.

Despite these promising results, several limitations remain. The current study evaluates the model only on the WM811K dataset, and further validation on more diverse wafer datasets and industrial scenarios is needed. In addition, the proposed framework focuses on wafer map classification and does not explicitly address defect localization or anomaly detection. Future work will explore extending the framework to more datasets and integrating more advanced representation learning strategies to further improve robustness and practical applicability.

## Figures and Tables

**Table 1:** Detailed Sample Distribution across Categories and Partitions (5% Labeling Ratio).

Defect Category	Total Samples	Training Set ($D_{l}$ + $D_{u}$)	Labeled Subset ($D_{l}$, 5%)	Validation Set
Center	4294	3006	150	429
Donut	555	389	19	55
Edge-Loc	5189	3632	182	519
Edge-Ring	9680	6776	339	968
Loc	3593	2515	126	359
Random	866	606	30	87
Scratch	1193	835	42	119
Near-full	149	104	5	15
None	147,431	103,202	5160	14,743
Total	172,950	121,065	6053	17,295

**Table 2:** Comparison of overall classification performance under the 5% labeled data setting.

Model	Precision (%)	Recall (%)	*F*1-Score (%)	Accuracy (%)
Supervised-CNN	79.21 ± 0.85	79.39 ± 0.92	79.30 ± 0.88	82.62 ± 0.74
PseudoLabel-CNN	84.61 ± 0.78	84.96 ± 0.81	84.78 ± 0.79	86.11 ± 0.65
MixMatch	85.32 ± 0.64	84.91 ± 0.68	85.11 ± 0.66	89.26 ± 0.55
FixMatch	90.61 ± 0.48	90.22 ± 0.55	90.41 ± 0.51	92.11 ± 0.43
MMVAE	91.24 ± 0.35	91.41 ± 0.38	91.32 ± 0.36	93.24 ± 0.30
MM-WAE	95.45 ± 0.21	95.88 ± 0.19	95.66 ± 0.22	96.11 ± 0.21

**Table 3:** Comparison of overall classification performance under the 10% labeled data setting on the WM811K dataset.

Model	Precision (%)	Recall (%)	*F*1-Score (%)	Accuracy (%)
Supervised-CNN	81.26 ± 0.85	81.45 ± 0.92	81.35 ± 0.88	83.12 ± 0.74
PseudoLabel-CNN	82.36 ± 0.78	82.77 ± 0.81	82.56 ± 0.79	84.15 ± 0.65
MixMatch	89.11 ± 0.54	88.75 ± 0.62	88.93 ± 0.58	91.32 ± 0.45
FixMatch	92.07 ± 0.38	92.54 ± 0.45	92.30 ± 0.41	94.75 ± 0.33
MMVAE	94.24 ± 0.25	94.41 ± 0.28	94.32 ± 0.26	95.84 ± 0.20
MMWAE (Ours)	96.11 ± 0.15	96.23 ± 0.18	96.17 ± 0.15	97.12 ± 0.12

**Table 4:** Ablation study of different modality combinations in MM-WAE on the WM811K dataset.

Model	Precision (%)	Recall (%)	*F*1-Score (%)	Accuracy (%)
MMWAE S	91.45	92.26	91.85	93.65
MMWAE SF	93.62	93.84	93.73	95.63
MMWAE ST	92.02	91.96	91.99	94.96
MMWAE	96.11	96.23	96.17	97.11

## Data Availability

The original contributions presented in this study are included in the article. Further inquiries can be directed to the corresponding author.
